# *Mycobacterium tuberculosis* GrpE, A Heat-Shock Stress Responsive Chaperone, Promotes Th1-Biased T Cell Immune Response via TLR4-Mediated Activation of Dendritic Cells

**DOI:** 10.3389/fcimb.2018.00095

**Published:** 2018-03-27

**Authors:** Woo Sik Kim, In Duk Jung, Jong-Seok Kim, Hong Min Kim, Kee Woong Kwon, Yeong-Min Park, Sung Jae Shin

**Affiliations:** ^1^Department of Microbiology, Institute for Immunology and Immunological Diseases, Brain Korea 21 PLUS Project for Medical Science, Yonsei University College of Medicine, Seoul, South Korea; ^2^Department of Biotechnology, Advanced Radiation Technology Institute, Korea Atomic Energy Research Institute, Jeongeup, South Korea; ^3^Lab of Dendritic Cell Differentiation and Regulation, Department of Immunology, College of Medicine, Konkuk University, Chungju, South Korea

**Keywords:** *Mycobacterium tuberculosis*, GrpE, dendritic cell, TLR4, Th1 polarization, immune response

## Abstract

*Mycobacterium tuberculosis* (Mtb), the causative agent of tuberculosis, is an extremely successful pathogen with multifactorial ability to control the host immune response. Insights into the Mtb factors modulating host response are required for the discovery of novel vaccine antigen targets as well as a better understanding of dynamic interactions between the bacterial factors and host cells. Here, we exploited the functional role of Mtb GrpE, a cofactor of heat-shock protein 70 (HSP70), in promoting naïve CD4^+^/CD8^+^T cell differentiation toward Th1-type T-cell immunity through interaction with dendritic cells (DCs). GrpE functionally induced DC maturation by up-regulating the expression of cell surface molecules (CD80, CD86, and MHC class I and II) and production of several pro-inflammatory cytokines (TNF-α, IL-1β, IL-6, and IL-12p70) in DCs. These effects of GrpE in DC activation were initiated upon binding to Toll-like receptor 4 (TLR4) followed by activation of downstream MyD88-, TRIF-, MAPK-, and NF-κB-dependent signaling pathways. GrpE-activated DCs displayed an excellent capacity to effectively polarize naïve CD4^+^ and CD8^+^ T cells toward Th1-type T-cell immunity with the dose-dependent secretion of IFN-γ and IL-2 together with increased levels of CXCR3 expression. Notably, GrpE-stimulated DCs induced the proliferation of GrpE-specific Th1-type effector/memory CD4^+^/CD8^+^CD44^high^CD62L^low^ T cells from the spleen of Mtb-infected mice in a TLR4-dependent manner. Collectively, these results demonstrate that GrpE is a novel immune activator that interacts with DCs, in particular, via TLR4, to generate Th1-biased memory T cells in an antigen-specific manner. GrpE may contribute to the enhanced understanding of host-pathogen interactions as well as providing a rational basis for the discovery of new potential targets to develop an effective tuberculosis vaccine.

## Introduction

Tuberculosis (TB) caused by *Mycobacterium tuberculosis* (Mtb) remains a serious global health problem as one of the top 10 causes of death worldwide in the twenty-first century (Small, 2009). Host immune responses play a crucial role in both detrimental and protective immunity against Mtb (Cooper, 2009; Kleinnijenhuis et al., 2011). In general, the Th1 type T cell response induced by Mtb antigens (Ags) is thought to be central to the protective immunity against Mtb infection (Cooper, 2009). Thus, isolation and characterization of Mtb Ags are necessary to clarify the host-pathogen interactions and to develop an effective vaccine and diagnostic Ags.

Although macrophages are thought to be the primary intracellular niche of Mtb, the host initial T cell response is dependent on the activation of dendritic cells (DCs) (Cooper, 2009). DCs are characterized as professional Ag-presenting cells that are important in bridging innate and adaptive immunity (Mihret, 2012). As a hallmark in TB, it has been suggested that Mtb likely subverts CD4 T-cell immunity by modulating DC functions leading to the initiation of T cell responses (Wolf et al., 2007; Gallegos et al., 2008; Cooper, 2009). In other words, early activation and migration of DCs toward draining lymph nodes, together with induction of T cells, are vital in the early protective immune response against Mtb infection (Cooper, 2009). These observations suggest that a mycobacterial Ag that elicits effective T-cell immunity through DC activation is a promising target in development of effective vaccine for TB. In fact, administration of DCs treated with BCG or pulsed with Mtb-specific Ags provided remarkable protection in a mouse model against virulent Mtb infection (Choi et al., 2017). Various mycobacterial Ags that trigger a Th1-type T cell immune response via the activation of DCs have been described (Byun et al., 2012b; Kim et al., 2015). However, little is known about their detailed molecular mechanism involved in initiating the immune response. This lack of knowledge has driven the continual identification of Ags that generate protective Th1-type T cell immunity. Novel immunogenic Ags are required for advancements including vaccine development and diagnostic techniques for Mtb infection.

Accumulating evidence suggests that pattern recognition receptors of DCs strive to promote innate immunity by mediating the secretion of diverse cytokines once the DCs encounter Mtb-associated Ags. The DC receptors ultimately contribute to adaptive immunity through up-regulating co-stimulatory molecules and major histocompatibility class (MHC) molecules, supporting the development of Mtb-specific Th1 responses (Cooper, 2009; Kleinnijenhuis et al., 2011; Mihret, 2012). Among these pattern recognition receptors, toll-like receptors (TLRs) play a pivotal role in the early innate immune response via the detection of characteristic molecular signatures carried by invading microorganisms (Kleinnijenhuis et al., 2011). Several experimental studies have revealed the important role of TLRs in Mtb protection and pathogenesis. Importantly, TLR2, TLR4, and TLR9 are all involved in the recognition of Mtb. The interactions between these TLRs and Mtb can induce the expression of adhesion molecules, cytokines, and chemokines, and lead to the recruitment of various immune cells, such as DCs and macrophages, to Mtb-infected sites (Mortaz et al., 2015). However, in TLR2-deficient mice, increased bacterial load, defective granulomatous response, and chronic pneumonia have been demonstrated upon aerosol infection with viable Mtb (Drennan et al., 2004). An additional study suggested that TLR4 signaling in Mtb protection appears to be required to control the local growth and disseminated Mtb infection from the lungs (Abel et al., 2002). Based on these studies, TLRs are likely essential for the initiation of host defenses against Mtb infection.

Recently, several studies reported that distinct mycobacterial components induce a TLR-dependent maturation and activation of DCs (Chen et al., 2004; Bansal et al., 2010; Byun et al., 2012a). Several mycobacterial proteins activate DCs to drive either a Th1 or a Th2 immune response via the TLR2/TLR4 or TLR2 pathway, respectively, indicating Ag selectivity effects in DCs (Bansal et al., 2010; Byun et al., 2012a). Among many Ags of Mtb interacting with DCs, many mycobacterial heat-shock proteins (HSPs) modulate host immune response by manipulating diverse TLRs, in particular, the interaction with DCs (Silva, 1999).

In general, under certain conditions of stress, all prokaryotic and eukaryotic cells constitutively express HSPs, which are highly conserved intracellular proteins (Zugel and Kaufmann, 1999b). Several studies have identified HSPs as targets of immune responses during mycobacterial infection (Zugel and Kaufmann, 1999a; Lewthwaite et al., 2001). Most of the focus has been on Mtb HSPs 60, 65, and 70 in the pathogenesis of TB because of their immunogenicity and ability to broadly activate immune cells (Zugel and Kaufmann, 1999a,b; Lewthwaite et al., 2001). Among the mycobacterial HSPs, Mtb HSP70 is the most well-known Ag liking innate and adaptive immune responses with potent adjuvant activity (Harmala et al., 2002). In Mtb, the HSP70 operon consisting of the *dnaK*-*grpE*-*dnaJ* (*rv0350*-*rv0351*-*rv0352*) genes is co-expressed and negatively regulated by the repressor *hspR* (*rv0353*) (Bandyopadhyay et al., 2012). GrpE derived from Mtb is a well-known HSP70 cofactor, which functions as a molecular chaperon and plays an important role in strengthening the survival of Mtb during cellular stresses, such as heat-shock and hypoxia (Stewart et al., 2002). GrpE is a constitutive Ag of HSP70 and is equally expressed in Mtb for protein synthesis, holding, trafficking, and degradation (Buck et al., 2007). GrpE has been identified in the culture supernatant, membrane protein fraction, and whole cell lysates of Mtb H37Rv (de Souza et al., 2011). The common roles of HSP70 and GrpE in the virulence, detoxification, and adaptation in Mtb growth have recently been demonstrated (Raman et al., 2004). Although the role of HSP70 in the immune response to Mtb infection has been studied extensively, relatively little attention has been given to its cofactor, GrpE, especially with respect to its immunological functions.

In the present study, we investigated the role of GrpE in the immune response, in particular, its interaction with DCs. A better understanding of Mtb GrpE and its role in the host immune response may aid in the rational design of more effective control strategies, including multi-antigenic Mtb subunit vaccines. Thus, we attempted to provide insights into the molecular basis of Mtb GrpE function by showing that GrpE activates DCs. Gaining a better understanding of the factors that contribute to protective host responses to Mtb infection will aid in the discovery of novel Ags to improve TB vaccine development.

## Materials and methods

### Animals

Female, specific pathogen-free (SPF), 6–7-week-old C57BL/6J (H-2K^b^ and I-A^b^), BALB/c (H-2Kd and I-A^d^) mice were purchased from Japan SLC, Inc. (Shizuoka, Japan). Age- and gender-matched ovalbumin (OVA)-specific T cell receptor (TCR) transgenic mice, TLR2 knockout (K/O) mice (B6.129-Tlr2tm1Kir/J), and TLR4 K/O mice (C57BL/10ScNJ) in a C57BL/6J background were purchased from the Jackson Laboratory (Bar Harbor, ME). The animals were fed a sterile commercial mouse diet and provided with water *ad libitum*. Mice infected with Mtb H37Rv were housed under adequate conditions in a BL-3 biohazard animal barrier facility at the Avison Biomedical Research Center, Yonsei College of Medicine. The animal experiments complied with the Ethics Committee and Institutional Animal Care and Use Committee of Yonsei University Health System (Permission no.: 2016-019).

### Bacteria culture and infection

Mtb H37Rv (ATCC 27294) was obtained from the International Tuberculosis Research Centre (Masan, South Korea). Mtb H37Rv was cultured aerobically at 37°C in Middlebrook 7H9 broth supplemented with 0.02% glycerol and 10% OADC for 4 weeks. Mtb H37Rv was incubated until mid-log phase in 7H9-OADC medium. For the animal infection studies, BALB/c mice were aerogenically infected with Mtb H37Rv, as previously described (Byun et al., 2012a). Briefly, Mtb H37Rv-infected mice were placed in the inhalation chamber of an airborne infection apparatus (Glas-Col, Terre Haute, IN) for a predetermined dose exposure of Mtb H37Rv for 60 min. After a 1-d exposure, Mtb H37Rv numbers were counted. Approximately 200 CFU of viable Mtb H37Rv were delivered into the lungs of Mtb H37Rv-infected mice. To conduct the mixed lymphocyte reactions, 8 week's post-infection, Mtb H37Rv-infected mice spleens were collected after euthanization.

### Cloning and purification of recombinant GrpE

*grpE* (*rv0351*) was amplified from genomic DNA of Mtb H37Rv by polymerase chain reaction (PCR). The *grpE* primer was: 5′-CGCCATATGGTGACGGACGGAAATCAAAAGC-3′ (forward primer; the *Nde* I site is underlined) and 5′-CCCAAGCTTACTGCCCGACGGTTCTGATTC-3′ (reverse primer; the *Hind* III site is underlined). The sequences of *grpE* in the pGEM-T Easy Vector plasmid (after PCR-amplified full-length open reading frame (ORF) of *grpE* was inserted) were confirmed using T7-specific primers. Finally, plasmids containing recombinant *grpE* were transferred into *E. coli* BL21 (DE3). Recombinant GrpE was expressed after induction with 1 mM isopropyl-β-d-thio-galactoside (IPTG) at 37°C for 12 h. After disruption of the bacteria by sonication, the GrpE proteins were purified using a nickel-nitrilotriacetic acid (Ni-NTA) column and polymyxin B-agarose (Sigma-Aldrich, St. Louis, MO) as previously described (Byun et al., 2012a). Finally, endotoxin content was analyzed using an LAL assay (Lonza, Basel, Switzerland) and was <11 pg/mL (<0.1 UE/mL) in the purified GrpE.

### Antibodies and reagents

For the generation of bone marrow-derived DCs (BMDCs), recombinant mouse granulocyte-macrophage colony stimulating factor (GM-CSF) and recombinant mouse interleukin-4 (IL-4) were purchased from JW CreaGene (Gyeonggi, South Korea). For cell viability analyses, fluorescein isothiocyanate (FITC)-annexin V/propidium iodine (PI) kits were obtained from eBioscience (San Diego, CA). Lipopolysaccharide (LPS, from *E. coli* O111:B4: Invivogen, San Diego, CA) was used as a positive control for the measurement of DC maturation. An endotoxin filter (END-X) and an endotoxin removal resin (END-X B15) were acquired from Associates of Cape Cod (East Falmouth, MA). Peptron was used to synthesize the OVA_257–264_ peptide (SIINFEKL) and OVA_323–339_ peptide (ISQAVHAAHAEINEAGR) in the T cell proliferation assay. For western blotting, anti-phosphorylated extracellular signal-regulated kinase (anti-p-ERK1/2), anti-ERK1/2, anti-p-c Jun, N terminal kinase (JNK), anti-JNK, anti-p-p38, anti-p38, anti-nuclear factor kappa B (NF-κB, p65), anti-p-IκB-α, and anti-IκB-α antibody (Ab) were purchased from Santa Cruz Biotechnology, Inc. (Dallas, TX). Horseradish peroxidase (HRP)-conjugated anti-mouse IgG Ab and anti-rabbit Abs were purchased from Calbiochem (San Diego, CA). Anti-β-actin and anti-Lamin B Abs were purchased from Sigma-Aldrich. For flow cytometry analysis, fluorescein isothiocyanate (FITC)-conjugated Abs to CD4, CD11c, CD62L and interferon-gamma (IFN-γ), APC-conjugated Abs to IL-12p70, Alexa647-conjugated Abs to CCR3 and CD3, Alexa700-conjugated Abs to CD3, PerCP-Cy5.5-conjugated Ab to CD4^+^ and CD8^+^, phycoerythrin (PE)-conjugated Abs to IL-4 and IFN-γ, CCR7, CD80, CD86, MHC class I, MHC class II, CD44, and CXCR3, PE-Cy7-conjugated Ab to IL-2 were purchased from eBioscience. For the cytokine analysis, IL-6, IL-1β, tumor necrosis factor-alpha (TNF-α), IL-2, IL-4, and IFN-γ Enzyme-linked Immunosorbent Assay (ELISA) kits were purchased from eBioscience, and IL-12p70 and IL-10 ELISA kits were purchased from BD Biosciences (San Diego, CA).

### Generation and culture of DCs

BMDCs were prepared and cultured as previously described (Byun et al., 2012a). To obtain highly purified populations for subsequent analyses, cells cultured for 8 days were stained with bead-conjugated anti-CD11c Ab and separated by positive selection through LS MACS columns according to the manufacturer's instructions (Miltenyi Biotec, Bergisch Gladbach, Germany). The purity of cells selected by anti-CD11c beads was > 90%.

### Assessment of surface molecules on DCs

After 24 h of GrpE treatment of DCs, markers of DC maturation including CCR7, CD80, CD86, MHC class I, and MHC class II were detected by fluorochrome-conjugated Abs to CCR7, CD80, CD86, and MHC class I and II. Moreover, CD11c^+^ expressed on all DCs was stained with anti-CD11c Ab. Positive cells that stained with both PE- and FITC-conjugated antibody were analyzed using a FACSCalibur flow cytometer (BD Biosciences, Franklin Lakes, NJ).

### Antigen uptake capacity

GrpE-treated DCs were cultured in the presence of 1 mg/mL dextran at 37°C and at 4°C (the negative control). DCs stained with anti-CD11c (PE) were analyzed using a FACSCalibur flow cytometer (BD Biosciences) to investigate antigen uptake.

### DC migration ability by GrpE

To evaluate the responsiveness of GrpE- or LPS-treated DCs to CCL19, an *in vitro* chemoattraction assay was performed in 24-well Transwell chambers (5 μm; Costar, Corning, NY). CCL19 (300 ng/mL) diluted in serum-free RPMI 1640 medium were added to lower chambers in a volume of 0.6 mL. DCs (1 × 10^5^ cells in 0.1 mL) resuspended in serum-free RPMI 1640 medium were deposited in the upper chambers. Migration of cells to the lower chamber of each well occurred during the 3-h incubation at 37°C. The numbers of migrating DCs in the lower chambers were counted over a 60-s period using a FACSCalibur flow cytometer (BD Biosciences).

### Analysis of peptide-MHC class complexes formation on DCs

The Eα_44–76_ peptide (RLEEFAKFASFEAQGALANIAVDKANLDVMKKR; the underlined sequence binds to MHC-II), OVA_257–264_ (SIINFEKL; the sequence binds to MHC-I) and Eα_52–68_ peptide (ASFEAQGALANIAVDKA; the sequence binds to MHC-II) were synthesized at 95% purity (Biorex, Budapest, Hungary). For analysis of peptide-MHC-I complex formation, the DCs were treated with 10 μg/mL GrpE in presence of 500 μg/mL OVA protein or OVA_257–264_ peptide (positive control). For analysis of peptide-MHC-II complex formation, the DCs were treated with or without 10 μg/mL GrpE in presence of 25 μg/mL Eα_44–76_ peptide or Eα_52–68_ peptide (positive control). After 24 h incubation, each cell was harvested and washed with phosphate buffered saline (PBS) twice and stained with anti-CD11c, anti-mouse OVA_257–264_ peptide bound to H-2Kb (PE, eBioscience) or anti-mouse Eα_52–68_ peptide bound to I-Ab (FITC, eBioscience) for 15 min at room temperature (RT). The stained cells were analyzed using FACSverse flow cytometer (BD Biosciences, Santa Clara, CA) using FlowJo software (BD Biosciences). Analysis of peptide-MHC-I complex formation was determined according to a procedure reported by Vander Lugt et al. (2014).

### LPS removal from GrpE preparation and LPS decontamination through polymyxin B binding, boiling, and proteinase K treatment

For pretreatment with polymyxin B (PMB), LPS and GrpE were incubated in a medium containing 10 μg/mL of PMB (Sigma-Aldrich) for 1 h at RT. For heat-denaturation, LPS or GrpE was incubated at 100°C for 30 min. For digestion of proteinase K (PK), LPS or GrpE were treated along with 10 μg/mL soluble PK for 1 h at 37°C. After 24 h of antigen treatment, DC maturation was analyzed based on TNF-α and IL-6 levels using cytokine-specific ELISAs (eBioscience and BD Biosciences).

### Cytotoxic analysis

After 24 h of antigen treatment, antigen-treated cells were washed and stained by FITC-Annexin V/PI. Thereafter, apoptosis and necrosis of DCs were analyzed using a FACSCalibur flow cytometer (BD Biosciences).

### Immunoprecipitation

DCs were stimulated with 10 μg/mL GrpE for 6 h at 37°C. After incubation, the cells were washed twice with PBS and lysed using an immunoprecipitation lysis buffer (Pierce, Rockford, IL). Total cell lysates were precleared for 2 h at 4°C overnight on a rotor with protein A or G Sepharose beads. GrpE and TLR2- and TLR4-associated proteins were incubated for 1 h at 4°C with control Abs (anti-rat IgG as control Ab for anti-TLR2 and TLR4, and anti-mouse IgG as control Ab for anti-His mAb), and then immunoprecipitated via incubation with protein A or G Sepharose for 24 h at 4°C. All control Abs were purchased from Santa Cruz Biotechnology, Inc. (Dallas, TX). The beads were harvested and washed twice with PBS. Finally, beads were boiled in 5X sample buffer for 5 min and then western blotting was performed. Each membrane was probed with anti-TLR2, -TLR4, or -His monoclonal Abs (mAbs), as designated.

### Western blot and nuclear extract preparation

After culture of DCs for 6 days, 10 μg/mL GrpE was treated for various times. The cells were harvested and washed twice with PBS. The washed DCs were lysed with RIPA buffer (Pierce). Immunoblotting was performed as previously described (Byun et al., 2012a). Nuclear extracts from cells were isolated using a Cellytic nuclear extraction kit (Sigma-Aldrich) according to the manufacturer's protocol.

### Treatment of DCs with pharmacological inhibitors of signaling pathways

All of the pharmacological inhibitors for MAPKs and NF-κB signaling were obtained from Calbiochem (San Diego, CA). Dimethyl sulfoxide (DMSO, Sigma-Aldrich) was added to the cultures at 0.1% (v/v) as a solvent control. DCs were pre-incubated with MAPK and NF-κB signaling inhibitors for 1 h at 37°C, and then treated with GrpE (10 μg/mL) for 24 h at 37°C. Inhibition was performed using the following inhibitors and concentrations; U0126 (10 μM), SP600125 (20 μM), SB203580 (20 μM), and Bay11-7082 (20 μM).

### Confocal laser scanning microscopy (CLSM)

DCs (1 × 10^5^ cells per well) were incubated on poly-L-lysine-coated glass coverslips for 24 h at 37°C and then treated with GrpE (10 μg/mL) for 1 h at 37°C. The cells were fixed in 4% paraformaldehyde, permeabilized in 0.1% Triton X-100, and blocked with 2% bovine serum albumin (BSA) in PBS containing 0.1% Tween-20 (PBS/T) for 2 h. Next, anti-his or anti-p65 mAbs in a 2% BSA/PBS/T solution were incubated for 2 h at RT. The cells were washed twice with PBS/T and then incubated with FITC-conjugated secondary Ab in the dark for 1 h. Finally, cells were stained with 4′,6-diamidino-2-phenylindole (DAPI, 1 μg/mL) for 10 min at RT. Cell morphology and fluorescence intensity were analyzed using a confocal laser microscope (LSM510, Carl Zeiss, Jena, Germany).

### T cell proliferation assay

OVA-specific CD4^+^ and CD8^+^ T cells (2 × 10^6^ cells per well) isolated from splenocytes of OT-I and OT-II mice using a MACS column were stained with carboxyfluorescein succinimidyl ester (CFSE, 1 μm) and co-cultured with DCs (2 × 10^5^ cells/well) treated with GrpE (10 μg/mL) in the presence of OVA peptides including OVA_323–339_ and OVA_257–264_. After co-culture for 3 days, T cells were stained with fluorochrome-conjugated Abs to CD4, CD8, CCR3, or CXCR3 and analyzed by flow cyetry. Supernatants were collected to measure the levels of IFN-γ, IL-2, and IL-4 using ELISA kits.

### Analysis of the activation of effector/memory T cells

Responder T cells, which participate in allogeneic T cell reactions, were isolated using anti- with bead-conjugated anti-CD4 and -CD8 Abs (Miltenyi Biotec) from the splenocyte population of Mtb H37Rv-infected BALB/c mice 8 weeks post-infection. DCs (2 × 10^5^ cells/well, C57BL/6J background) generated by Wild-type (WT), TLR2 K/O, and TLR4 K/O mice were induced with GrpE (10 μg/mL) for 24 h at 37°C. GrpE-treated DCs were co-cultured with isolated CD4 and CD8 T cells at a DC:T cell ratio of 1:10. After 3 days of co-culture, the cells were further stained with fluorochrome-conjugated Abs to CD4, CD8, CD62L, and CD44, and analyzed by flow cyetry using a FACSCanto device (BD Biosciences). Immunoblotting using T-bet and GATA-3 Abs was performed to investigate T-bet and GATA-3 expression in T cells isolated from the co-culture experiment.

### Cytokine measurement

Cytokine levels were measured using quantitative ELISA kits, following the manufacturer's instructions (eBioscience and BD Biosciences). Cytokine levels were estimated by measuring absorbance at 450 nm with a microplate reader.

### Statistical analyses

Experiments featured a minimum of three repetitions with repeatable results. The levels of significance for comparison between samples or groups were established by an unpaired *t*-test or one-way ANOVA followed by Tukey's multiple comparison test distribution using statistical software (GraphPad Prism Software, version 5.0; GraphPad Software, San Diego, CA). The data in the graphs are expressed as the mean ± standard deviation (*SD*). Each *p*-value (^*^p < 0.05, ^**^p < 0.01, ^***^p < 0.001) was considered to be statistically significant.

## Results

### Recombinant GrpE induces maturation and activation of DCs

**Figure 1:**
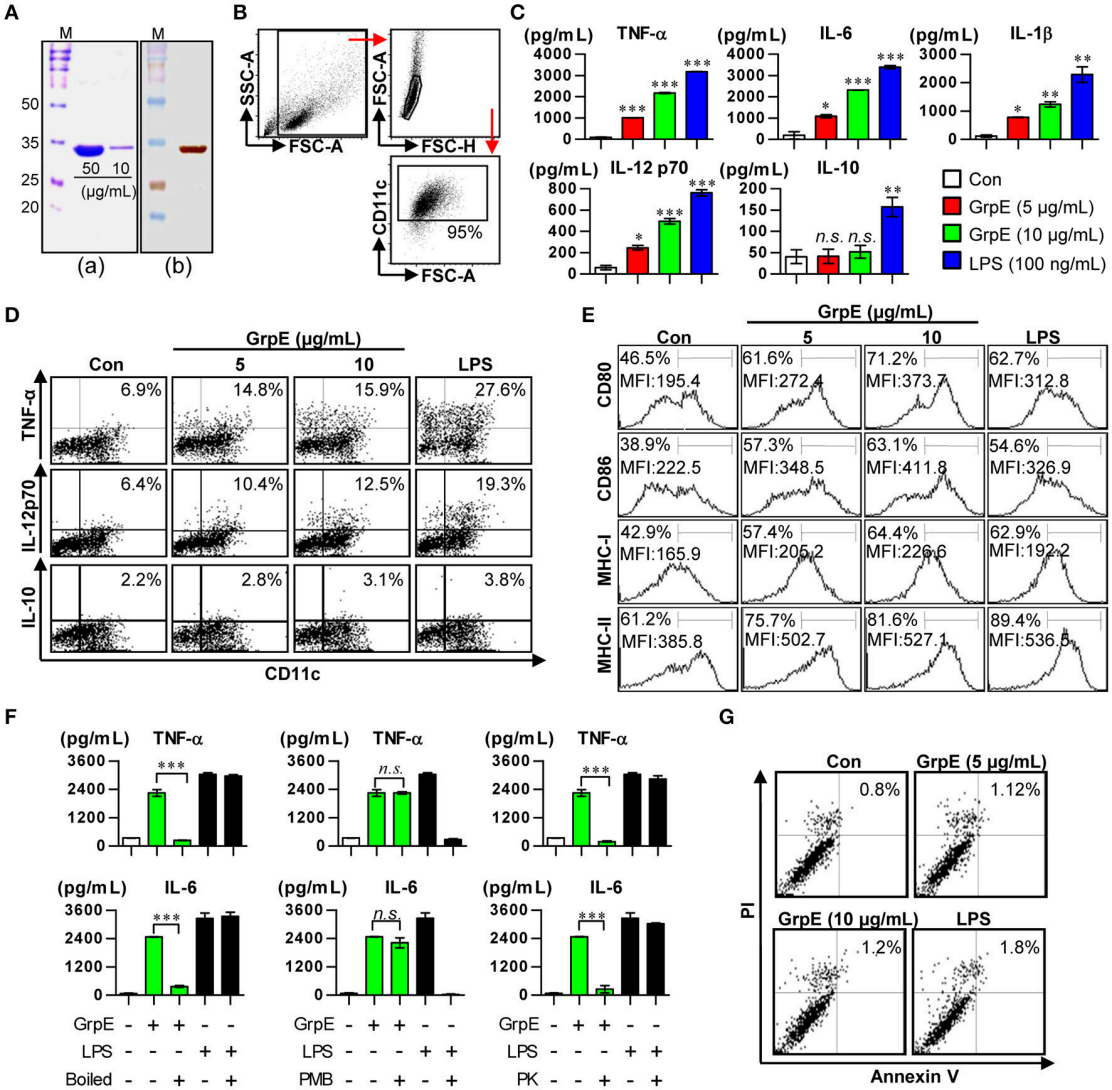
Preparation of recombinant GrpE and analysis of primary characteristics of DC maturation by GrpE treatment. **(Aa)** SDS–PAGE analysis of purified GrpE by Ni-NTA (M, markers; Bands, GrpE loading amount). **(Ab)** Western blot analysis of purified GrpE (10 μg) using mouse anti-His Ab. **(B)** Gating strategy of purified CD11c^+^ DCs. **(C)** Cytokine production by GrpE-treated DCs. DCs were generated by stimulating magnetic bead-purified immature DCs with LPS (100 ng/mL), GrpE (5 or 10 μg/mL) for 24 h, followed by ELISA for IL-12p70, TNF-α, IL-6, IL-1β, and IL-10 production. **(D)** Analysis of TNF-α, IL-12p70, and IL-10 expression in DCs by intracellular cytokine staining after 12 h at 37°C in the presence of GolgiPlug (1 μg/mL). One representative plot from three independent experiments is shown. **(E)** CD11c^+^ DCs were stained with anti-CD80, CD86, and MHC class I and II mAb and the expression levels of surface molecules were measured. One representative plot from three independent experiments is shown. **(F)** GrpE and LPS were heated for 1 h at 100°C (left panels) or digested with PMB (middle panels) or PK (right panels) before adding it to DC culture, as described in the section Materials and Methods. After 24 h treatment, TNF-α and IL-6 levels in the supernatant of DCs were analyzed using ELISA. These results are expressed as the mean ± *SD* (*n* = 3 samples) of representative result in three experiments (^*^p < 0.05, ^**^p < 0.01, or ^***^p < 0.001). **(G)** DCs treated with GrpE or LPS were harvested 24 h later. The DCs were stained with anti-CD11c, annexin V, and PI and analyzed by flow cytometry. One representative plot from three independent experiments is shown. Con denotes untreated DCs.

In this study, we intended to determine whether Mtb GrpE, whose immunological function remains unknown, especially by interacting with DCs. First, GrpE was expressed as a fusion protein with a 6x His-tag in *E. coli* BL21. GrpE was primarily prepared as a soluble form. SDS-PAGE revealed that the molecular weight of the protein was approximately 32.0 kDa (Figure 1Aa). Purification of the protein was ultimately confirmed by Western blotting (Figure 1Ab). After successful purification of GrpE protein, we analyzed whether GrpE induces DC maturation. We first focused on cytokine secretion of cultured BMDCs (Figure 1B) activated by GrpE. As shown in Figure 1C, GrpE increased pro-inflammatory cytokines (IL-12p70, TNF-α, IL-6, and IL-1β) in a dose-dependent manner, while levels of the anti-inflammatory cytokine IL-10 were not statistically different from negative controls (untreated DCs). This result was confirmed by intracellular staining, which showed that GrpE induced TNF-α and IL-12p70, but not IL-10 (Figure 1D). Based on these results, we hypothesized that GrpE induces the expression of surface molecules on DCs. Therefore, the expression of co-stimulatory molecules (CD80 and CD86) and MHC class molecules on DCs were analyzed by flow cytometry. GrpE significantly induced the expression of these surface molecules in a dose-dependent manner (Figure 1E). Next, treatment with PK or PmB, and heat-denaturation were carried out to analyze LPS contamination (Figure 1F). PK pretreatment and heat-denaturation abrogated the ability of GrpE to trigger DC maturation. PmB treatment did not affect the ability of GrpE, whereas the ability of LPS was significantly inhibited by PmB treatment. We also identified that these effects of GrpE were not due to cytotoxicity, because there were no remarkable differences in the percentage of dead cells upon GrpE stimulation (Figure 1G). The findings indicated that the intact GrpE induced the maturation of DCs independent of contaminating endotoxins and cell death.

### GrpE increases migration and Ag-presenting capability of DCs with reduced antigen uptake

**Figure 2:**
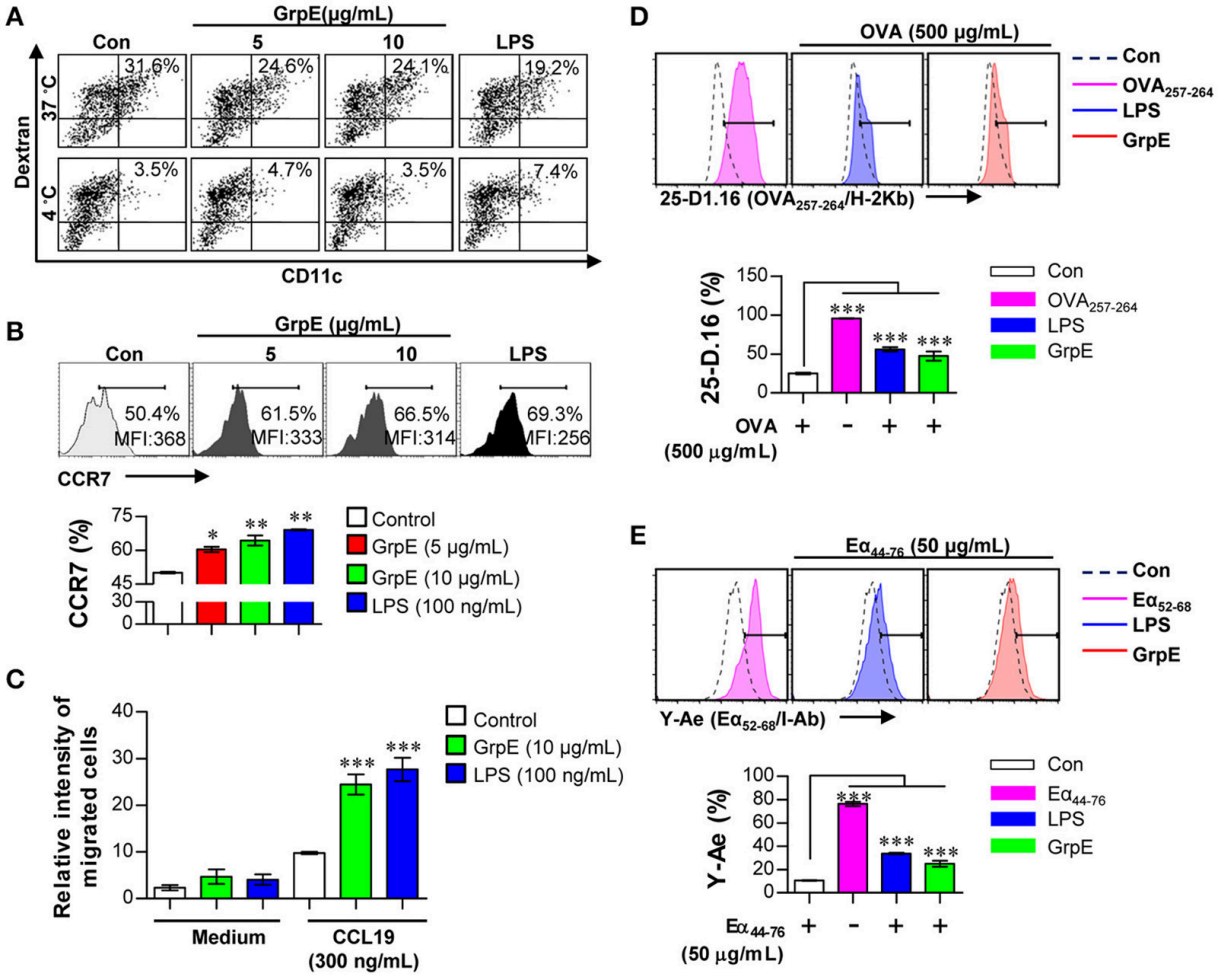
Ag uptake, migration, and Ag presentation capability of DCs by GrpE treatment. **(A–C)** DCs were treated with GrpE or LPS for 24 h. **(A)** Cells were incubated with dextran-FITC at 37 and 4°C for 30 min, and assessed by flow cytometry. The percentages of dextran^+^CD11c^+^ cells are indicated. One representative plot from three independent experiments is shown. **(B)** CCR7 expression in DCs (on gated CD11c^+^) was analyzed by flow cytometry. **(C)** DCs were treated with GrpE and the relative intensity of migrated cells was identified through CCL19. Bar graphs display mean ± *SD* (*n* = 3 samples) and statistical significance (^*^p < 0.05, ^**^p < 0.01, or ^***^p < 0.001) is shown for treatments compared to non-treated DCs (Control). **(D**,**E)** DCs were treated with LPS or GrpE, and then pulsed with OVA protein or Eα_44–76_ peptide. After 24 h, cells were stained with anti-CD11c, anti-25-D1.16, or anti-Y-Ae mAbs. Histogram and bar graphs for expression of OVA_257–264_/H-2Kb **(D)** and Eα_52–68_/I-Ab complexes **(E)** are displayed. OVA_257–264_ or Eα_52–68_ peptide (each peptide; 2 μg/mL concentration) was used as a positive control for antigen presentation. Histogram data are representative result of three experiments. Bar graph data is expressed as the mean ± *SD* (*n* = 3 samples); ^***^p < 0.001; Con denotes untreated DCs.

We observed the maturation of DCs by examining the secretion of pro-inflammatory cytokines and the expression of surface molecules induced by GrpE. Because activated DCs lose their endocytic ability, this was used as a characteristic feature in the maturation of DCs. We assessed the reduced endocytic capacity after the treatment of DCs with various doses of GrpE in the presence of dextran-FITC. Cells positive for CD11c and dextran were evaluated by flow cytometry. As displayed in Figure 2A, a markedly lower percentage of active cells was detected in GrpE-treated DCs compared with untreated DCs (control, Con). The same experiment was performed at 4°C as the negative control to demonstrate that the findings reflected endocytosis and not dextran penetration. We hypothesized that GrpE-mediated maturation of DCs induces CCR7 expression, which in turn enhances DC migration in response to CCL19. To explore this hypothesis, we measured the expression of CCR7 after GrpE stimulation. As shown in Figure 2B, the expression of CCR7 was enhanced in GrpE-treated DCs, similar to LPS-treated DCs used as a positive control. To investigate the effects of GrpE on the migration of DCs in response to CCL19, we next analyzed the migratory capacity of DCs *in vitro* using Transwell chambers. GrpE increased the migratory capacity of DCs in response to CCL19 compared with that in the medium control (Figure 2C). To determine the exogenous Ag-presenting ability for DCs, we analyzed peptide-MHC class complex formation using anti-25-D1.16 mAb, which reacts with MHC-I associated OVA_257–264_, and anti-Y-Ae mAb, which recognizes the Eα_52–68_ peptide-MHC-II complex. OVA_257–264_/MHC-I complexes (Figure 2D) and Eα_52–68_/MHC-II complexes (Figure 2E) were frequently observed in GrpE-treated DCs compared with non-treated DCs. These results indicate that GrpE can induce DC maturation via the induction of exogenous Ag-presenting ability as well as other maturation-related events.

### GrpE induces DC activation through the interaction with TLR4

**Figure 3:**
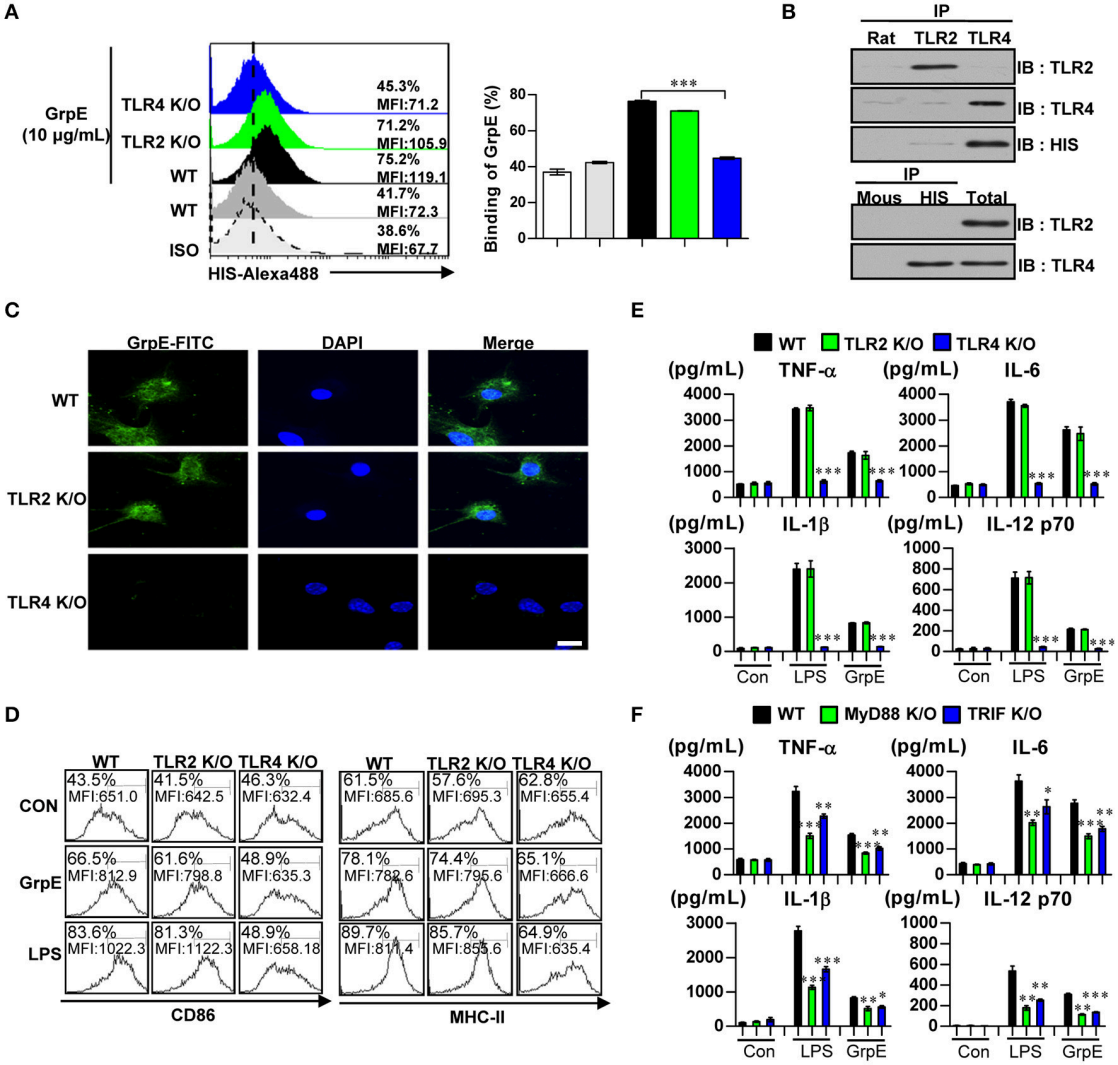
Induction of DC maturation by GrpE via MyD88/TRIF-dependent TLR4 signaling pathway. **(A)** DCs derived from WT-, TLR2 K/O-, and TLR4 K/O-mice were treated with GrpE (10 μg/mL) for 1 h and stained with an Alexa488-conjugated anti-His mAb. The percentage of positive cells is shown in each panel. Bar graph data are expressed as the mean ± *SD* (*n* = 3 samples); ^***^p < 0.001. **(B)** DCs were treated with GrpE for 6 h. The cells were harvested and cell lysates were immunoprecipitated with anti-rat IgG, anti-mouse IgG, anti-His, anti-TLR2, or anti-TLR4 mAbs, and proteins were visualized by immunoblotting with anti-His, anti-TLR2, or anti-TLR4 Abs. One representative plot from three independent experiments is shown. **(C)** Fluorescence intensities of anti-GrpE bound to GrpE-treated DCs. DCs derived from WT-, TLR2 K/O-, and TLR4 K/O-mice were treated with GrpE (10 μg/mL) for 1 h, fixed, and stained with DAPI and FITC-conjugated anti-GrpE Ab (Scale bar: 10 μm). One representative plot from three independent experiments is shown. **(D)** DCs purified from WT-, TLR2 K/O-, and TLR4 K/O-mice were treated with GrpE and LPS, respectively. After 24 h, we measured the expression of CD86 and MHC class II on DCs by flow cytometry. Data are representative result of three experiments. **(E)** IL-12p70, TNF-α, IL-6, and IL-1β in supernatants of GrpE- or LPS-treated DCs cultured from WT-, TLR2 K/O-, and TLR4 K/O-mice was measured with ELISA. **(F)** DCs derived from WT-, MyD88 K/O-, and TRIF K/O-mice were treated with GrpE and LPS for 24 h. The amount of IL-12p70, TNF-α, IL-6, and IL-1β in culture medium of GrpE-treated DCs was measured by ELISA. All data are expressed as the mean ± *SD* (*n* = 3 samples) of representative result in three experiments and statistical significance (^*^p < 0.05, ^**^p < 0.01, or ^***^p < 0.001) vs. GrpE-treated WT-DC groups (Black bar). Con denotes untreated DCs.

TLRs are pivotal in the innate immune response. Importantly, evidence indicates that the recognition of Mtb infection involves TLR2 or TLR4 (Kleinnijenhuis et al., 2011). We analyzed whether GrpE could be recognized by, and act through, TLRs in DCs. To identify GrpE interaction specificity with TLRs on DCs, WT-DC, TLR2 K/O-DC, and TLR4 K/O-DCs were treated with GrpE. Alexa488-conjugated anti-GrpE mAb was used to detect GrpE on the cell surface. Anti-GrpE bound selectively to the cell surface of WT-DCs and TLR2 K/O-DCs, while no interaction was detected between GrpE and the cell surface of TLR4 K/O-DCs (Figure 3A). To identify the interaction between GrpE and TLR4, we performed immunoprecipitation studies with TLR2 or TLR4 in DCs. These showed that GrpE was bound to TLR4, but not TLR2 (Figure 3B). This result was also confirmed by confocal microscopy. As expected, the co-localization of GrpE and TLR2 K/O-DCs, but not TLR4 K/O-DCs, was also observed (Figure 3C). To confirm the ability of GrpE to activate DCs via TLR4, we analyzed the expression of surface molecules (Figure 3D) and production of pro-inflammatory cytokines (Figure 3E) in GrpE-treated WT-, TLR2 K/O-, and TLR4 K/O-DCs. When surface molecules and the levels of pro-inflammatory cytokines of GrpE-treated DCs were assessed, the results indicated that the immune response was induced in WT- or TLR2 KO-DCs, but not in TLR4 KO-DC. Thus, these effects markedly declined in TLR4 KO-DC, showing that GrpE is an agonist for TLR4 in DCs.

The myeloid differentiation primary response 88 (MyD88) protein is involved in the signaling of almost all TLRs, except for TLR3. Additionally, TIR-domain-containing adapter-inducing interferon-β (TRIF) is essential for TLR4-mediated activation (Kleinnijenhuis et al., 2011). These TLR signaling adaptor molecules play a pivotal role controlling the various signaling pathways that are activated by TLR4. To investigate the importance of the MyD88- and TRIF-dependent pathways in GrpE-induced cytokine production by DCs, we analyzed the cytokine production in DCs generated by WT-, MyD88 K/O-, and TRIF K/O-mice. GrpE-induced productions of TNF-α, IL-6, IL-1β, and IL-12p70 were significantly reduced in the absence of MyD88 and TRIF (Figure 3F). These results suggested that MyD88 and TRIF are crucial for an optimal GrpE-induced cytokine response. These findings suggest that GrpE induce DC maturation in a TLR4-dependent manner, causing increased expression of cell-surface molecules and pro-inflammatory cytokines.

### Mitogen-activated protein kinases (MAPKs) and NF-κB signaling pathways participate in GrpE-mediated DC activation

**Figure 4:**
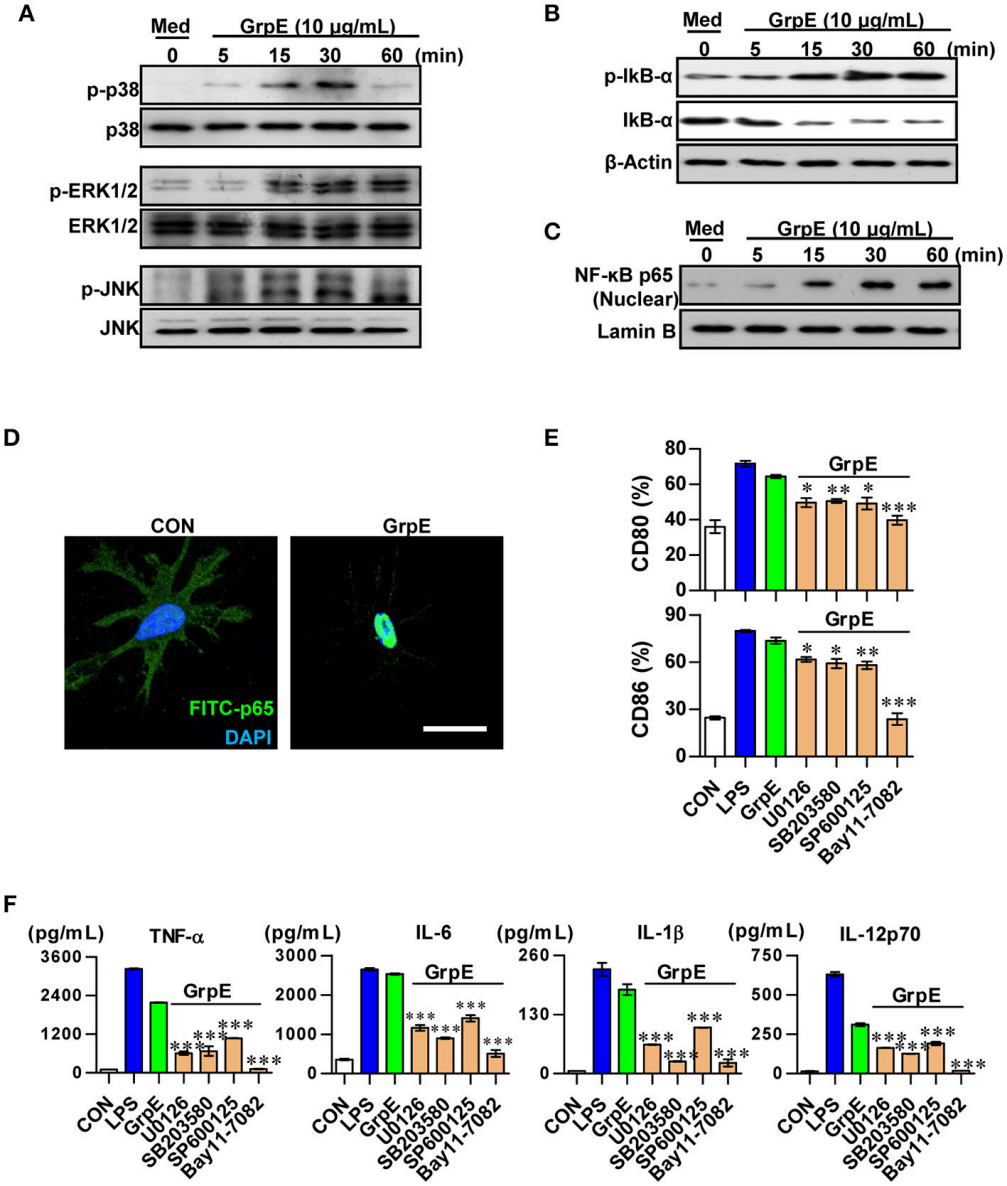
Involvement of the MAPKs and NF-κB pathways in GrpE-induced DC activation. Western blot analysis of the cytoplasmic fraction **(A**,**B)** for phospho-p38 (p-p38), p38, phospho-ERK1/2 (p-ERK1/2), ERK1/2, phospho-JNK (p-JNK), JNK, phosphor-IκB-α, and p-IκB-α from whole cell extracts and nuclear fraction **(C)** p65 NF-κB. β-actin and Lamin B served as loading controls from cytoplasmic and nuclear fractions, respectively. **(D)** Immunofluorescence subcellular localization of the p65 protein. Representative results of three independent experiments are shown. **(E**,**F)** DCs were pretreated at 37°C for 1 h with the pharmacological inhibitors SB203580 (p38), U0126 (ERK1/2), SP600125 (JNK), Bay 11-0782 (NF-κB), or dimethylsulfoxide (vehicle control) and then exposed to 10 μg/mL GrpE for 24 h. **(E)** GrpE and pharmacological inhibitors-treated DCs were stained with anti-CD80 and anti-CD86 and analyzed by flow cytometry. Bar graphs show the percentage for each surface molecule on CD11c^+^ cells. The mean ± *SD* (*n* = 3 samples) of representative of three independent experiments. **(F)** The amounts of TNF-α, IL-6, IL-1β, and IL-12p70 in the culture media were measured by ELISA. The mean ± *SD* (*n* = 3 samples) are shown representative of three independent experiments and statistical significance (^*^p < 0.05, ^**^p < 0.01, or ^***^p < 0.001) is shown for treatments compared to GrpE only-treated DCs (Green bars). Con denotes untreated DCs.

MAPKs and NF-κB pathways are important in the activation of DCs. They promote the expression of certain surface molecules and promote the transcriptional activity of cytokine genes (Boisleve et al., 2005). Therefore, we examined the phosphorylation of ERK1/2, JNK, and p38 upon treatment of DCs with GrpE. GrpE led to the phosphorylation of these proteins (Figure 4A). In addition, GrpE promoted the phosphorylation and degradation of IκB-α, leading to the nuclear translocation of p65 (Figures 4B–D). Next, to elucidate the functional roles of these signaling molecules induced by GrpE, we used specific kinase inhibitors and analyzed GrpE-induced pro-inflammatory cytokine production and co-stimulatory molecule expression. Notably, we found that GrpE-induced the expression of CD80 and CD86 from DCs (Figure 4E), and the production of TNF-α, IL-6, IL-1β, and IL-12p70 was significantly abolished by these pharmacological inhibitors through the inhibition of the NF-κB and MAPK signaling pathways (Figure 4F). From these findings, we suggest that the NF-κB and MAPK signaling pathways are essential for DC maturation induced by GrpE.

### GrpE-activated DCs induce T cell proliferation and Th1 polarization

**Figure 5:**
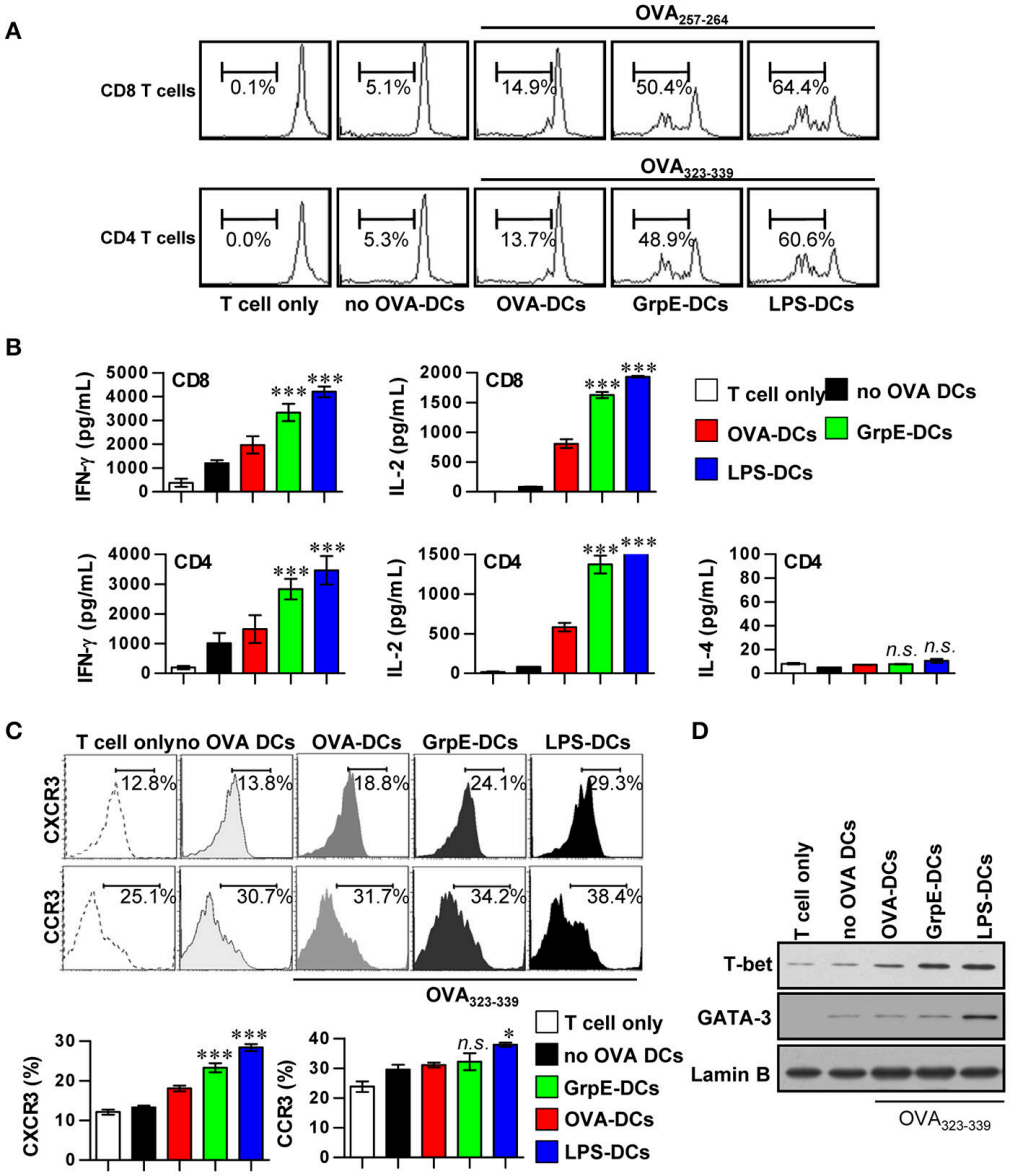
Induction of T cell proliferation and Th1 polarization by GrpE-treated DCs. **(A)** Transgenic OVA-specific CD8^+^ T cells and CD4^+^ T cells were isolated, stained with CFSE, and co-cultured for 96 h with DCs treated with GrpE (10 μg/mL) and LPS (100 ng/mL), then pulsed with 1 μg/mL OVA_257–264_ or OVA_323–339_ for OVA-specific T cells, respectively. T cells only and T cells co-cultured with untreated DCs (no OVA-DCs) served as controls. The proliferation of CD4^+^ and CD8^+^ T cells was then assessed by flow cytometry. This data is shown representative of three independent experiments**. (B)** At 96 h after co-culture, IFN-γ, IL-2, and IL-4 levels in culture supernatants were analyzed by ELISA. **(C)** T cells were stained with anti-CXCR3 mAb or anti-CCR3 mAb. The percentage of positive cells is shown for each panel. Histograms and bar graphs show CXCR3^+^ or CCR3^+^ T cells in the OVA-specific CD4^+^ T cells. The mean ± *SD* (*n* = 3 samples) is shown representative of three independent experiments and statistical significance (^*^p < 0.05 or ^***^p < 0.001) is shown for treatments when compared to the appropriate controls (T cell/OVA-pulsed DCs, red bars). Treatments with no significant effect are indicated as *n.s*. **(D)** T-bet and GATA-3 expression in the OVA-specific CD4^+^ T cells was assessed by immunoblotting using specific anti-T-bet and anti-GATA-3 mAbs. Representative results of three independent experiments are shown.

We assessed the effects of GrpE on increased T cell proliferation using OVA-specific T cells from OT-I and OT-II transgenic mice. The mature DCs induced by GrpE pulsed with OVA_257–264_ or OVA_323–339_ were co-cultured with either CFSE-labeled OVA-specific CD4^+^ or CD8^+^ T cells. CD4^+^ and CD8^+^ T cell proliferation was induced using OVA_257–264_- or OVA_323–33_-treated DCs in the presence of GrpE and compared to the same T cells co-cultured with OVA_257–264_- or OVA_323–33_-treated DCs without GrpE (Figure 5A). The production levels of IFN-γ and IL-2 by naïve CD4^+^ and CD8^+^ T cells stimulated by GrpE-treated DCs (GrpE-DCs) was significantly higher (*P* < 0.05–0.01), whereas IL-4 was not detected (Figure 5B). We next inquired the expression of CXCR3 and CCR3, which have been proposed as Th1 and Th2 cell polarization markers (Yamamoto et al., 2000). CD4^+^ T cells stimulated by GrpE-treated DCs in the presence of OVA_323–339_ displayed a significant increase in the expression of CXCR3 compared to CD4^+^ T cells stimulated with OVA_323–339_-treated DCs only. GrpE did not influence the expression of CCR3, while LPS-treated DCs induced the expression of CCR3 (Figure 5C). In addition, CD4^+^ T cells stimulated with GrpE-treated DCs induced the expression of T-bet (a Th1-specific transcription factor), whereas there was no expression of GATA-3 (a Th2-specific transcription factor) detected (Figure 5D). These results indicate that GrpE contributed to the naïve T cell proliferation toward a Th1 phenotype.

### GrpE-activated DCs induces expansion of Ag-specific effector/memory T cell subpopulations during Mtb infection in a TLR4-dependent manner

**Figure 6:**
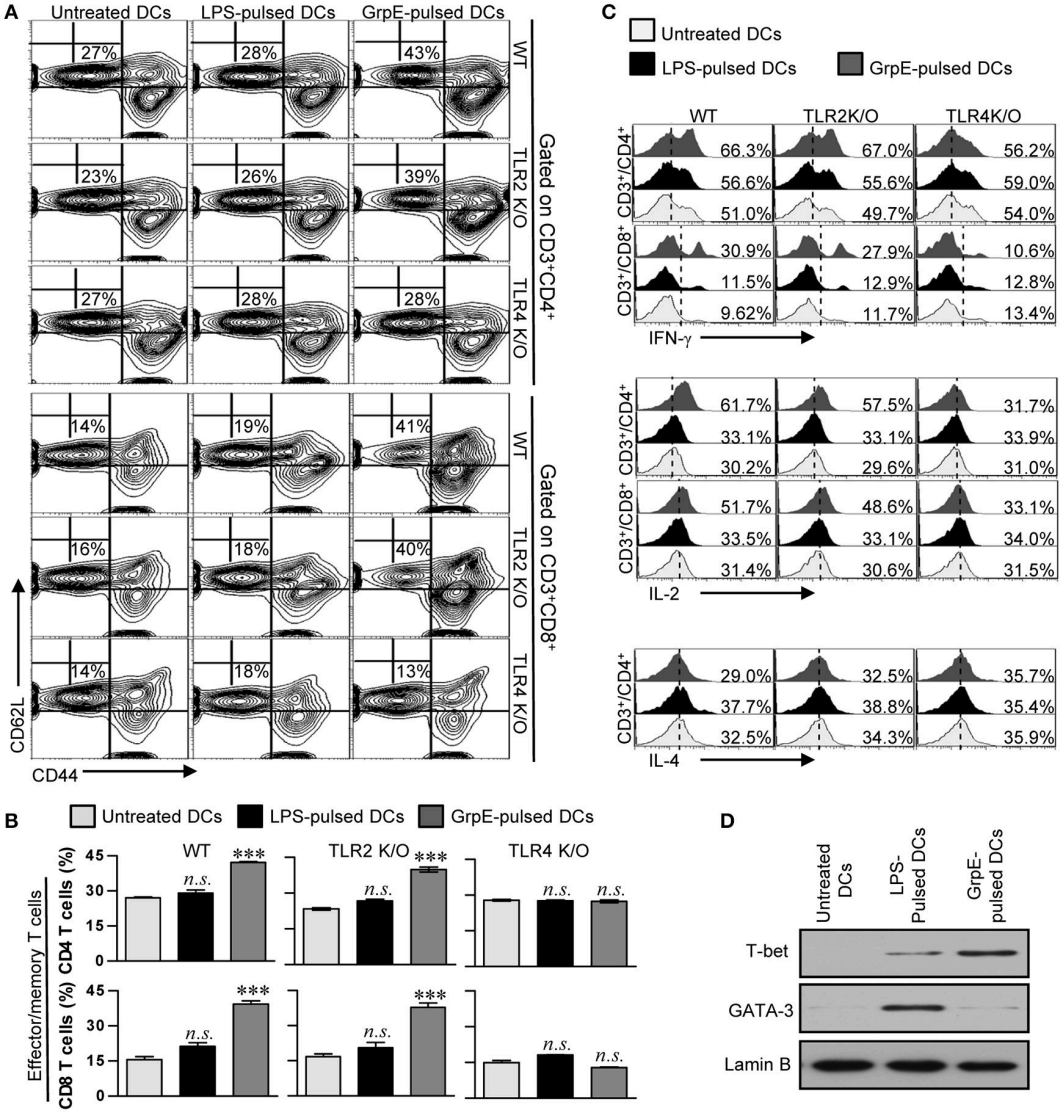
Induction of the effector/memory T cell proliferation and a Th1 response by GrpE-activated DCs via TLR4-dependent DC maturation. **(A**,**B)** WT-, TLR2 K/O-, and TLR4 K/O-DCs were treated with 10 μg/mL GrpE for 24 h. The GrpE-treated DCs were washed and co-cultured with allogeneic T cells (splenocytes from BALB/c mice infected with Mtb H37Rv) at DC to T cell ratios of 1:10 for 3 days. T cells were stained with anti-CD4, anti-CD8, anti-CD62L, and anti-CD44 mAbs. Contour **(A)** and bar graphs **(B)** show CD62L^+^CD44^+^ T cells. **(B)** Bar graphs show the percentages (mean ± *SD, n* = 4 samples) for CD44^+^CD62L^−^ T cells from representative result of three independent experiments. Statistical significance (^***^p < 0.001) is indicated for treatments compared to untreated DCs, and no significant effect is indicated as *n.s*. **(C)** After 12 h of co-culture, IFN-γ, IL-2, and IL-4 expression levels in CD3^+^CD4^+^ and CD3^+^CD8^+^ cells were analyzed in T cells co-cultured with untreated DCs, GrpE-pulsed DCs, and LPS-pulsed DCs by intracellular staining for the presence of GolgiPlug. Results are representative of three independent experiments. **(D)** WT-DCs were cultured with GrpE stimulation for 24 h. The GrpE-treated DCs were co-cultured for 3 days with splenic T cells from Mtb H37Rv-infected mice and then suspended in lysis buffer. T-bet and GATA-3 expression in cell lysate was assessed by immunoblotting using specific anti-T-bet and anti-GATA-3 Abs. Western blot results are representative of three independent experiments.

Th1-based DCs induced by Mtb Ag have the potential to polarize naïve T cells toward a Th1-phenotype, as well promote the expansion and activation of Ag-specific memory T cells (Snijders et al., 1998). To assess whether the maturation of DCs by GrpE is reflected in their ability to specifically stimulate CD4^+^ and CD8^+^ T cells from the spleens of Mtb H37Rv-infected mice at 8 week's post-infection, we examined the expression of the memory T cell markers CD62L and CD44 on the surface of CD4^+^ and CD8^+^ T cells using flow cytometry. Splenocytes of Mtb-inoculated mice were co-cultured with GrpE-treated DCs derived from WT, TLR2 K/O, and TLR4 K/O mice. GrpE-treated WT and TLR2 K/O-DCs explicitly promoted the formation of CD44^high^CD62L^low^, which is an effector/memory T cell type, compared to untreated DCs or LPS-treated DCs, while this outcome of GrpE was abolished in TLR4 K/O-DCs (Figures 6A,B). We also found that the proportions of IFN-γ^+^ and IL-2^+^ cells among the CD4^+^ and CD8^+^ T cell populations stimulated with WT- or TLR2 K/O-DCs in the presence of GrpE were higher compared to cells stimulated with untreated DCs or LPS-treated DCs. These populations were not detectable in T cells stimulated with TLR4 K/O-DCs. In addition, the proportions of IL-4^+^ cells in the CD4^+^ cell population stimulated with GrpE-pulsed DCs or LPS-pulsed DCs remained at baseline levels (Figure 6C). Furthermore, T cells stimulated with GrpE-treated DCs induced the expression of T-bet, whereas no expression of GATA-3 was detected (Figure 6D). These findings suggested that DCs matured with GrpE promote a TLR4-dependent expansion and activation of effector/memory T cells, which drives Th1 polarization in Mtb H37Rv-infected mice.

## Discussion

Inside host cells, including macrophages and DCs, Mtb encounters many stress conditions (Zugel and Kaufmann, 1999b; Kleinnijenhuis et al., 2011; Mihret, 2012). The ability of Mtb to survive under oxidative stress *in vivo* is an important aspect of its pathogenesis. Many studies have sought to understand the survival of Mtb under stress conditions such as heat, reduced oxygen or hypoxia, nutrient starvation, and downshift in pH (Farhana et al., 2010; Giffin et al., 2016; Sukheja et al., 2017). Mtb responds to the stress conditions by genome wide transcriptional changes, including the induction of a transient and read expression of a well-conserved set of genes (Flentie et al., 2016). In this context, HSPs are essential molecular chaperones for maintaining cellular functions during normal as well as stress conditions (Zugel and Kaufmann, 1999a). Several recent studies clarified the immunological functions of mycobacterial HSPs. For example, Mtb HSP70 contributes to enhanced MHC-I/-II antigen processing, whereas Mtb HSPX activates DCs, subsequently affecting CD8^+^ T cells (Tobian et al., 2004; Jung et al., 2014). Mtb HSP70 also reportedly increases in the levels of IL-1α, IL-1β, IL-6, TNF-α, and GM-CSF secreted by macrophages (Retzlaff et al., 1994). However, despite significant progress in understanding the pathophysiological and immunological functions of HSPs, the complete immunobiological significance of their cofactors remains unclear.

The *dnaK* operon is present in Gram-positive bacteria (Weng et al., 2001). The operon includes *grpE* and *dnaJ* and has been used to analyze the phylogenetic relationships among various organisms. Interestingly, multiple copies of *dnak* and *dnaJ* homologous genes are present in Mtb, while only a single gene copy of *grpE* is present (Cole et al., 1998; Weng et al., 2001; Lupoli et al., 2016). In addition, phylogenetic trees based on the sequence identity of GrpE among Gram-positive bacteria close agrees with that that obtained with 16S rRNA phylogeny, indicating that GrpE may be important in the evolutionary process and is highly conserved with high-G+C DNA *Mycobacterium* spp. (Ahmad et al., 2000). In addition, *grpE* has been demonstrated to be an essential gene by Himar1-based transposon mutagenesis in the H37Rv strain (Sassetti et al., 2003; Griffin et al., 2011). In fact, in addition to the constitutive overexpression of *grpE*, basal *grpE* expression is required because it is an essential factor for the survival and growth of Mtb, indicating that GrpE may be a good candidate vaccine Ag if it induces the appropriate anti-Mtb Th1 immune response. Overexpression of GrpE was identified from most prevalent clinical Mtb strains from Southern India under a hypoxic culture condition compared to aerated cultures, while other HSPs including HSPX, GroEL2, and GroES are expressed by the laboratory-adapted strain H37Rv. Thus, GrpE is predicted to be up-regulated during clinical Mtb infection and may be a potential immunogen (Devasundaram et al., 2016).

Memory Th1 cells are a keystone of the immune system that generate a protective immune memory response against Mtb infection and are a promising foundation of successful vaccination (Kaufmann, 2010). T cell immunity regulated by APCs is thought to be a major feature of protective immunity and most empirical approaches have focused on the ability of vaccine to induce a dominant T cell-mediated immune response (Wolf et al., 2007). Robust Th1 immune responses in Ag-specific memory T cells play an important role in the control of Mtb infection and are a key element in successful vaccines (Wolf et al., 2007; Kaufmann, 2010; Mihret, 2012). Several studies showed the signaling cascade via the TLR4-MyD88/TRIF-dependent pathways result in the induction of T cell proliferation and effector/memory T cells (Jin et al., 2012). Thus, TLR signaling plays an important part in vaccine development and can enhance the protective immune response against Mtb. Although Mtb TLR4 agonist is incompletely defined, recent studies have demonstrated that HSPs from mycobacteria bind to TLR2 and/or TLR4 (Bulut et al., 2005). In particular, Mtb-derived HSP70 was reported to induce the production of cytokines, such as IL-6, TNF-α, and IL-10, from DCs via TLR2 and TLR4, and to regulate the proliferation and differentiation of T cells (Wendling et al., 2000; Wieten et al., 2009).

Identification of Mtb TLR4 agonist is meaningful in two aspects. First, TLR4 participates in the initial recognition in the immune response to Mtb infection by recognizing Mtb Ags and influencing both innate and adaptive immunity (Means et al., 1999; Carmona et al., 2013). For example, *in vivo*, in a C3H/HeJ murine infection model deficient in TLR4 signaling pathway, a higher mycobacterial burden was evident in the lungs, spleen, and liver, and the survival rate of the TLR4-deficient mice was lower following infection compared with WT mice (Abel et al., 2002). Furthermore, Judith et al. reported TLR4 plays a protective role in pulmonary TB in mice (Branger et al., 2004). Results obtained using *in vitro* models have also demonstrated the importance of TLR4 in the recognition of Mtb Ags (Jang et al., 2017). Second, TLR4 agonists have attracted more attention recently as adjuvants for vaccines by virtue of their potential immunoregulatory effects (Wang et al., 2002; Liu et al., 2011). For example, an adjuvant function of the C-terminal portion of HSP70 or full-length HSP70 was shown to be responsible for stimulating Th1-polarizing cytokines in humans, indicating the potential adjuvant activity of TLR4 agonists derived from Mtb (Wang et al., 2002; Jung et al., 2011, 2014; Liu et al., 2011). Moreover, the use of Mtb heparin-binding hemagglutinin and the TLR4 agonist, HspX, in adjuvant treatment of cancer was recently reported. The signals, which are likely to be powerful immunopotentiators, are modulated by MyD88-and TRIF-dependent pathways mediated by these receptors (Jung et al., 2011, 2014). Therefore, the isolation and identification of novel Mtb Ags that interface with host TLR4 is crucial for the refinement of adjuvants and formulation of more competent vaccines.

The development of efficient vaccines and more influential therapies for TB requires a deeper comprehension of the overlapping networks of immune responses to immunogenic entities of Mtb, including the role of Mtb HSP-associated molecules, which is considered an integral part. We conclude that Mtb GrpE signals via TLR4 are followed by downstream signaling activation in a MyD88-, TRIF-, MAPKs and NF-κB-dependent manner. Our study thus demonstrates a major task for TLR4 signaling in response to GrpE produced by Mtb, and deepens our understanding of the molecular mechanisms modulating these responses. Elucidation of the molecular signaling mechanisms prompted by Mtb GrpE could help in the reasonable modeling of enhanced potent vaccines or of vaccine adjuvants to preserve the host from this exogenous invasion, and upgrading the immune responses against malignant tumors. Our findings highlight the molecular intercommunication between the heat-shock stress response Ag of Mtb and host immune cells, which is critical for the rational design of efficacious vaccines and for the understanding of Mtb pathogenesis. Head-to-head vaccine efficacy testing between HSP70 and GrpE against the highly virulent Mtb Beijing strain is underway.

## Author contributions

WK, IJ, and SS: Designed and performed experiment, analyzed data, and wrote the manuscript; J-SK, HK, Y-MP, and KK: Contributed to the discussion and interpretation of the results.

### Conflict of interest statement

The authors declare that the research was conducted in the absence of any commercial or financial relationships that could be construed as a potential conflict of interest.
